# Intestinal Microbiota and Weight-Gain in Preterm Neonates

**DOI:** 10.3389/fmicb.2017.00183

**Published:** 2017-02-08

**Authors:** Silvia Arboleya, Pablo Martinez-Camblor, Gonzalo Solís, Marta Suárez, Nuria Fernández, Clara G. de los Reyes-Gavilán, Miguel Gueimonde

**Affiliations:** ^1^Department of Microbiology and Biochemistry of Dairy Products, Instituto de Productos Lácteos de Asturias (CSIC)Villaviciosa, Spain; ^2^Geisel School of Medicine at Dartmouth, Dartmouth CollegeHanover (NH), USA; ^3^Universidad Autónoma de ChileSantiago, Chile; ^4^Pediatrics Service, Hospital Universitario Central de Asturias, Servicio de Salud del Principado de Asturias (SESPA)Oviedo, Spain; ^5^Pediatrics Service, Hospital de Cabueñes, Servicio de Salud del Principado de Asturias (SESPA)Gijón, Spain

**Keywords:** newborn, premature-infant, weight-gain, colonization, microbiota, probiotics

## Abstract

The involvement of the gut microbiota on weight-gain and its relationship with childhood undernutrition and growth has been reported. Thus, the gut microbiota constitutes a potential therapeutic target for preventing growth impairment. However, our knowledge in this area is limited. In this study we aimed at evaluating the relationship among early microbiota, growth, and development in preterm infants. To this end we assessed the levels of specific microorganisms by qPCR, and those of short chain fatty acids by mean of gas-chromatography, in feces from 63 preterm newborns and determined their weight-gain during the first months. The statistical analyses performed indicate an influence of the intestinal microbiota in weight-gain, with the levels of some microorganisms showing a significant association with the weight-gain of the infant. The levels of specific microbial groups during the first days of life were found to affect weight gain by the age of 1 month. Moreover, clustering of the infants on the basis of the microbiota composition at 1 month of age rendered groups which showed differences in weight z-scores. Our results suggest an association between the gut microbiota composition and weight-gain in preterm infants at early life and point out potential microbial targets for favoring growth and maturation in these infants.

## Introduction

Microbial colonization of the neonatal gut provides a stimulus necessary for the development of the intestine and the physiological homeostasis (Hooper and Macpherson, 2010; Sommer and Bäckhed, 2013). The early neonatal period represents the most important moment for this microbiota-induced maturation of the host, being a key determinant for later health (El Aidy et al., 2013). This neonatal colonization is affected by several factors such as gestational age, mode of delivery, antibiotic use, or feeding habits (Faa et al., 2013).

From the pioneering studies showing a reduction of Bacteroidetes and an increase of Firmicutes in obese animals (Ley et al., 2005), and the mechanistic works demonstrating the involvement of the gut microbiota in the regulation of food energy harvesting, fat storage and modulation of the endocrine function (Bäckhed et al., 2004; Cardinelli et al., 2015), the role of the microbiota in weight-gain has attracted increasing attention (Cardinelli et al., 2015). The modulation of the gut microbiota offers a potential therapeutic target for both, weight-management (Dror et al., 2017) and prevention of growth impariment (Blanton et al., 2016). Most data on the relationship between microbiota and weight-gain come from animal models or from studies in adults. Epidemiologycal data in infants are also available, evidencing associations between microbiota composition and later obesity (Koleva et al., 2015). However, these studies have focused on full-term infants and this interaction has not been explored in other populations, such as preterm babies, where weight-gain may be an indicator of maturation and positive outcome.

Preterm infants present an immature immune system and gut barrier, which leads to an increased disease risk. Moreover, the process of microbiota establishment is altered in these infants, who harbor more *Enterobacteriaceae* and potential pathogens and less commensals than full-terms (Arboleya et al., 2012, 2015). In these infants the nutritional goal is achieving a weight-gain and body composition approximating the fetal intrauterine growth (Brennan et al., 2016). For this reason, the adjustment of the preterm infant growth to the growth-tables, calculated from data on patients born at various gestational ages, is widely used to estimate newborn size and postnatal evolution (Fenton and Kim, 2013). This adjustment would be a positive nutritional outcome but it is difficult to achieve. Thus, developing strategies to enhance growth and maturation of preterm infants could be of clinical interest. Given the relationship between microbiota and weight-gain, the gut microbiota establishment process may constitute an adequate target.

The present study aims at evaluating the potential relationship between early microbiota development and weight-gain in preterm neonates.

## Materials and methods

### Subjects and samples

Sixty-three preterm infants born at gestational ages between 28 and 33 weeks were recruited at the Neonatology Units of Cabueñes Hospital and the University Central Hospital of Asturias (Northern Spain). None of the infants had necrotizing enterocolitis or culture positive early onset infection. All infants received mixed feeding (infant formula and some breast-milk administration during the study period). The infants were discharged from the hospital after an average stay of 38 days of hospitalization.

Fecal samples were collected at 2, 10, and 30 days-of-age and weights determined monthly up to 3 months. Weight data were used to calculate weight-gain at different sampling times and weight Z-scores were determined using the growth curves obtained by Fenton (Fenton and Kim, 2013). Fecal samples were immediately frozen and sent to the laboratory for analyses. The Regional Ethical Committee of Asturias Public Health Service approved the study and informed written consent was obtained from the parents.

### Intestinal microbiota and SCFA analyses

The absolute levels of the different bacterial populations analyzed, including the main representatives of the predominant phyla in the infant gut (*Bifidobacterium, Streptococcus, Staphylococcus, Enterococcus, Bacteroides*-group, *Enterobacteriaceae, Lactobacillus*-group, *Weissella*, and total bacteria), were determined by quantitative PCR using primers and conditions previously described (Arboleya et al., 2012). When a sample resulted negative for a certain microbial group, the value of the detection limit obtained for the corresponding primer pair (ranging between 10^3^ and 10^4^ cells per gram depending on the bacterial group) was assigned. For the preparation of standard curves, pure cultures of appropriate strains were used as previously reported (Arboleya et al., 2012).

Analysis of the main short-chain-fatty-acids (SCFA) (acetate, propionate, and butyrate) was carried out in supernatants of homogenized feces by using a 6890N gas chromatograph (Agilent Technologies Inc., Palo Alto, CA, USA) connected to a FID and a MS 5973N detectors (Agilent) as previously reported (Arboleya et al., 2012).

### Statistical analyses

Multiple mixed linear models were used to investigate the relationship between the microbial levels and fecal SCFA with weight-gain, adjusting by possible confounders. Backward stepwise analyses based on the Aikaike Information Criterion (AIC) were employed to determine whether the variables were included in predictive models. A forest plot was used in order to show the effect sizes in both the so-labeled univariate and the multivariate models (always adjusting by infant and gestational ages). With the goal of investigating different groups of subjects based on microbiota measures, a Euclidean cluster was performed. Dendrogram was used to determine the number of groups and standard robust Welch tests. Standard parametric robust Welch tests or non-parametric Kruskal-Wallis test, depending on the data distribution, was used to check equality among groups. Analyses were performed with R software, the conventional *p*-level of 0.05 was used in the interpretation of results.

## Results

The study included 28 males and 35 females (birth weights from 1,085 to 1,580 g). Twenty-two were vaginally delivered (VD) whilst 41 were born by caesarean section (CS). During the 3 months of duration of the study 35 of the infants received antibiotics at some time, most of them during the first month. The characteristics of the study population and the evolution of the intestinal microbiota in the total cohort and in the cohort subdivided according to delivery mode are reported in Supplementary Tables 1 and 2, respectively. In general, bacterial levels, and the concomitant production of SCFA, increased along the study, which is in good agreement with previously reported data (Arboleya et al., 2012, 2016).

**Figure 1:**
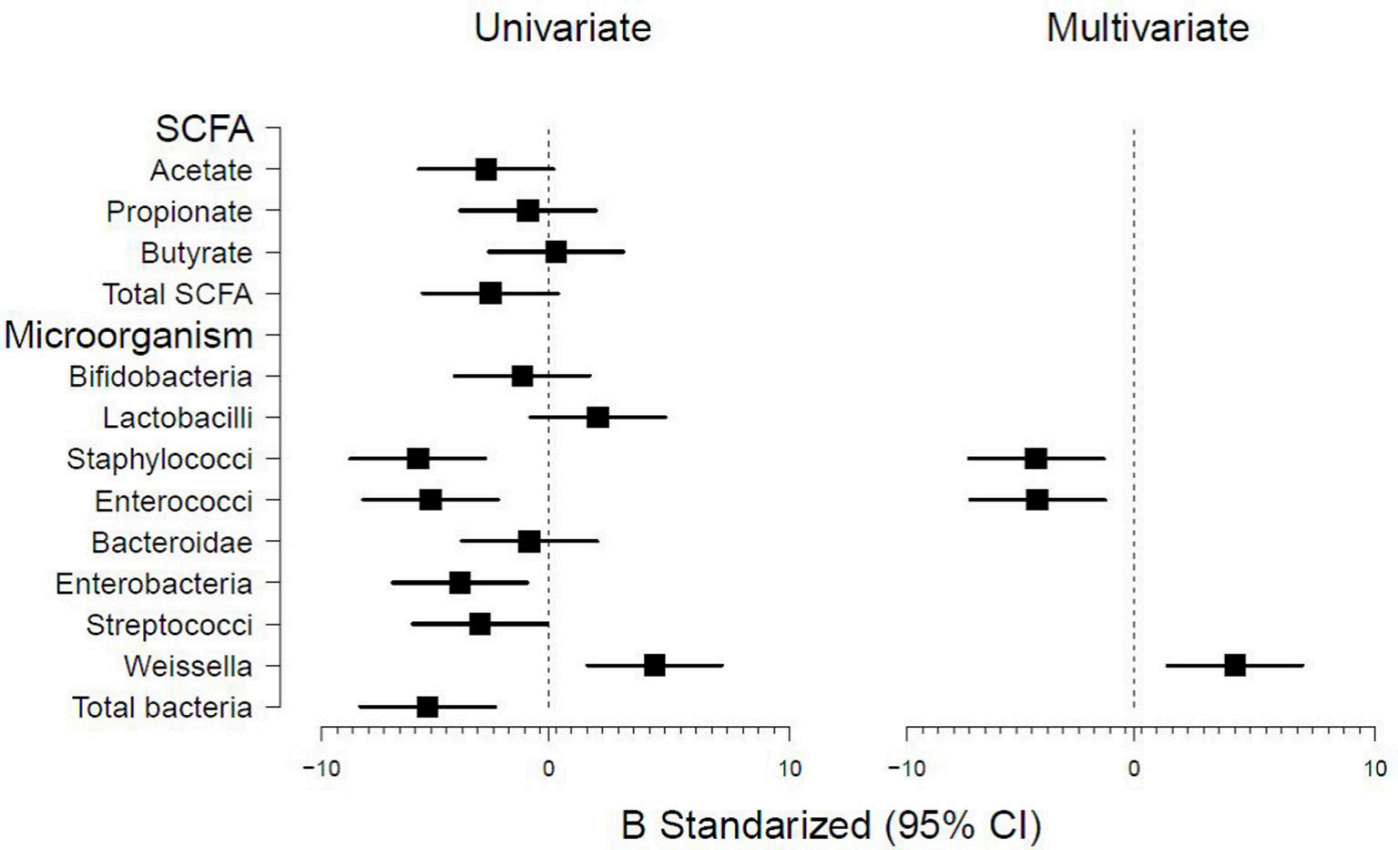
**Forest plot showing the microbiota-related variables measured and association with infant weight determined by univariate and multivariate generalized linear models**. Models were adjusted by infant and gestational age.

After the first few days, infants' weight increased, fitting well into a linear structure (Supplementary Figure 1). Initial exploratory analyses were conducted by using generalized mixed linear effects model with infant as random effect and gestational age as spline. The model showed a large effect of time (infant age) and gestational age, which together explained 80.5% of variance (determination coefficient, *r*^2^ = 0.805). Therefore, to further assess these associations the model was adjusted for these two variables and analyzed by multivariate regression to determine the effects of the microbiota-related variables. The results indicated an association of the intestinal microbiota with weight-gain in preterm infants (Figure 1). A significant effect of the levels of *Staphylococcus* (*p* < 0.001), *Enterococcus* (*p* < 0.001), *Enterobacteriaceae* (*p* = 0.011), *Streptococcus* (*p* = 0.047), *Weissella* (*p* < 0.001), and total bacteria (*p* = 0.002) was observed. In the full multivariate model (using backward elimination stepwise regression based on AIC) three variables remained significant; *Enterococcus* (*p* = 0.004), *Staphylococcus* (*p* = 0.005), and *Weissella* (*p* = 0.004) (Figure 1). The other microbial groups analyzed did not show any significant effect. Similarly, none of the SCFA analyzed showed a significant effect.

To evaluate the ability of the early microbiota-related variables for predicting weight-gain, the association of the microbiota variables at 2 and 10 days-of -age with the weight gained at 30 days of age (as percentage of birth-weight) was studied. Gestational age was found to be the major contributing factor, explaining a 55% of variance (*r*^2^ = 0.55). After adjusting for gestational age and birth weight (both together explaining 56% of variance), the levels of *Enterobacteriaceae* at 2 and 10 days-of-age, *Streptococcus* and total bacteria levels at 2 days, and those of *Bacteroides-group* at 10 days were found to have a significant effect upon weight gain (Table 1), increasing a 17.6% the percentage of variance explained, up to a total of 73.5%. None of the other microorganisms was found associated with weight-gain at 1 month, although a trend was observed for bifidobacterial levels (estimate 3.23 ± 1.64, *p* = 0.054).

**Table 1:** **Microbiota-related variables at 2 and 10 days of infant age that were associated with weight-gain at 30 days of life (measured as percentage with regard to birth-weight)**.

**Variable**	**Multivariate**^a^		
	**Estimate**	**Standard error**	***p***
*Enterobacteriaceae* at 2 days	15.892	2.701	< 0.001
*Streptococcus* at 2 days	7.080	2.217	0.003
*Total bacteria* at 2 days	−19.159	3.288	< 0.001
*Enterobacteriaceae* at 10 days	−3.316	1.174	0.007
*Bacteroides-group* at 10 days	5.234	1.982	0.011

a *Adjusted for gestational age and basal weight*.

Finally, the microbiota data obtained at 1 month of age were used for performing a cluster analysis, which allowed identifying three microbiota-driven infant groups (Supplementary Figure 2). These groups were not significantly different (*p* > 0.05) regarding gestational age, birth weight in grams or weight Z-score at birth (data not shown). However, infant weight at 3 months of age showed a statistically significant difference among clusters (*p* = 0.033). At this later time infant weight in group 3 (3003 ± 681 gr) was significantly lower than in the other two infant groups, that did not show statistically significant differences between them (3988 ± 1163 gr for group 1 and 3883 ± 1344 gr for group 2). Similarly, at 3 months of age Z-scores differed among groups (*p* = 0.013) with group 3 (−2.37 ± 2.24) differing significantly from groups 1 and 2 (−0.62 ± 1.08 and −0.90 ± 1.90, respectively).

When the statistical models used in this study were adjusted for the potential confounders “delivery mode” or “antibiotic exposure” significant effects were not observed, which indicates that these variables are not influencing the associations observed. Actually, in our study delivery mode did not show a large effect on the analyzed microorganisms, since only the *Bacteroides*-group was found to be statistically different between vaginal and C-section babies, with reduced levels in the latter group at 10 and 30 days of life (Supplementary Table 2).

## Discussion

An altered intestinal microbiota during the first year of life, with reduced levels of bifidobacteria and increased number of *Staphylococcus aureus*, has been observed in infants developing obesity later on (Kalliomäki et al., 2008). Particular microbial species, such as *Bacteroides fragilis*, have been related with higher BMI z-scores (Vael et al., 2011; Scheepers et al., 2014). However, this evidence is still limited and focuses in full-term infants and obesity prevention, whilst nothing is known on the relationship between microbiota and weight-gain in preterm infants.

Our results indicate an association between the intestinal microbiota and weight-gain. The levels of some microorganisms, such as *Staphylococcus* and *Enterococcus*, were negatively associated with weight-gain whilst others such as *Weissella* did it positively. This last microorganism belongs to the *Leuconostoccaceae* family from the *Lactobacillales* order (Fusco et al., 2015), being then related to *Lactobacillus*, some of whose strains were found to maintain growth of infant mice during chronic undernutrition (Schwarzer et al., 2016). Since lactobacilli are often used as probiotics, their ability, as well as that of non-pathogenic *Weissella* strains, to promote growth and maturation in preterm infants should be further explored.

Moreover early microbiota was associated with later weight-gain, suggesting a potential effect of the early microbiota in weight-gain in preterm infants. Especially, *Enterobacteriaceae* and *Streptococcus* levels at 2 days of age and *Bacteroides*-group levels at day 10 showed a positive association with weight gain at 1 month of age. To this regard, the species *B. fragilis* has been repeatedly associated to increased BMI (Vael et al., 2011; Scheepers et al., 2014) in infants, pointing out at these microorganisms as potential players in the association intestinal microbiota-weight gain. When infants were clustered on the basis of their microbiota at 30 days of age, differences among the clusters were obtained for infant weight and weight Z-scores at 3 months of age, again suggesting a relationship between microbiota and weight-gain. To this regard, certain oligosaccharides have been reported to promote a microbiota-dependent increase in body-mass (Charbonneau et al., 2016), and transplanting the microbiota of undernourished children into germ-free mice resulted in growth impairment (Blanton et al., 2016). Similar relationships between intestinal microbiota and growth have also been reported in infants (Gough et al., 2015), and delayed colonization by specific commensals has been related with altered adiposity (Dogra et al., 2015). All these reports suggest the involvement of the gut microbiota in infants' weight-gain and development.

Neither the delivery mode, nor the antibiotic exposure, showed a significant effect in any of the statistical models used in this study. These facts point out that these important potential confounders are not influencing the observed associations between microbiota and weight-gain. To this regard, in the present study the delivery mode was found to have a limited impact on intestinal microbial levels; *Bacteroides* was the only group significantly affected by delivery mode, presenting lower counts in babies born by C-section than in those vaginally delivered. The reduced levels observed for this microbial genus is in agreement with previous studies carried out in a similar preterm infants group (Arboleya et al., 2015). In relation to this, reduced levels of *Bacteroides* in full-term babies born by C-section have also been reported (Jakobsson et al., 2014), although in full-term babies the impact of C-section seem to be larger (Dominguez-Bello et al., 2010) than in our cohort of preterm infants.

Our results underline the interest of exploring the intestinal microbiota as a potential target for favoring growth and maturation in preterm infants. However, the meaning of weight-gain in terms of infant maturation, disease risk, and later weight is a matter of concern (Wang et al., 2016) that requires of further, larger, and longer observational and intervention studies.

## Author contributions

PM, GS, MS, NF, CD, and MG. designed the study; GS, MS, and NF recruited the infants and collected the data and samples; SA, CD, and MG. performed the microbiological analyses; PM, GS, and MG analyzed the data; all authors participated in the data interpretation, revised the manuscript and approved its final version. All authors agreed to be accountable for all aspects of the work in ensuring that questions related to the accuracy or integrity of any part of the work are appropriately investigated and resolved.

## Funding

This work was founded by the EU Joint Programming Initiative–A Healthy Diet for a Healthy Life (JPI HDHL, http://www.healthydietforhealthylife.eu/) and the Spanish Ministry of Economy and Competitiveness (MINECO) (Project EarlyMicroHealth). The Grant GRUPIN14-043 from “Plan Regional de Investigación del Principado de Asturias” is also acknowledged.

### Conflict of interest statement

The authors declare that the research was conducted in the absence of any commercial or financial relationships that could be construed as a potential conflict of interest.

## References

[B1] Arboleya S. Binetti A. Salazar N. Fernández N. Solís G. Hernández-Barranco A.. (2012). Establishment and development of intestinal microbiota in preterm neonates. FEMS Microbiol. Ecol. 79, 763–772. 10.1111/j.1574-6941.2011.01261.x 22126419

[B2] Arboleya S. Sánchez B. Milani C. Duranti S. Solís G. Fernández N.. (2015). Intestinal microbiota development in preterm neonates and effect of perinatal antibiotics. J. Pediatr. 166, 538–544. 10.1016/j.jpeds.2014.09.041 25444008

[B3] Arboleya S. Sanchez B. Solis G. Fernández N. Suárez M. Hernández-Barranco A. M.. (2016). Impact of prematurity and perinatal antibiotics on the developing intestinal microbiota: a functional inference study. Int. J. Mol. Sci. 17:649. 10.3390/ijms17050649 27136545 PMC4881475

[B4] Bäckhed F. Ding H. Wang T. Hooper L. V. Koh G. Y. Nagy A.. (2004). The gut microbiota as an environmental factor that regulates fat storage. Proc. Natl. Acad. Sci. U.S.A. 101, 15718–15723. 10.1073/pnas.0407076101 15505215 PMC524219

[B5] Blanton L. V. Charbonneau M. R. Salih T. Barratt M. J. Venkatesh S. Ilkaveya O.. (2016). Gut bacteria that prevent growth impairments transmitted by microbiota from malnourished children. Science 351:aad3311. 10.1126/science.aad3311 26912898 PMC4787260

[B6] Brennan A. M. Murphy B. P. Kiely M. E. (2016). Optimising preterm nutrition: present and future. Proc. Nutr. Soc. 75, 154–161. 10.1017/S0029665116000136 27032990

[B7] Cardinelli C. S. Sala P. C. Alvez C. C. Torrinhas R. S. Waitzberg D. L. (2015). Influence of intestinal microbiota on body weight gain: a narrative review of the literature. Obes. Surg. 25, 346–353. 10.1007/s11695-014-1525-2 25511750

[B8] Charbonneau M. R. O'Donnell D. Blanton L. V. Totten S. M. Davis J. C. Barratt M. J.. (2016). Sialylated milk oligosaccharides promote microbiota-dependent growth in models of infant undernutrition. Cell 164, 859–871. 10.1016/j.cell.2016.01.024 26898329 PMC4793393

[B9] Dogra S. Sakwinska O. Soh S. E. Ngom-Bru C. Brück W. M. Berger B. (2015). GUSTO study group. Dynamics of infant gut microbiota are influenced by delivery mode and gestational duration and are associated with subsequent adiposity. mBio 6:e02419 10.1128/mBio.02419-14 25650398 PMC4323417

[B10] Dominguez-Bello M. G. Costello E. K. Contreras M. Magris M. Hidalgo G. Fierer N.. (2010). Delivery mode shapes the acquisition and structure of the initial microbiota across multiple body habitats in newborns. Proc. Natl. Acad. Sci. U.S.A. 107, 11971–11975. 10.1073/pnas.1002601107 20566857 PMC2900693

[B11] Dror T. Dickstein Y. Dubourg G. Paul M. (2017). Microbiota manipulation for weight change. Microb. Pathog. [Epub ahead of print]. 10.1016/j.micpath.2016.01.002 26792677

[B12] El Aidy S. Hooiveld G. Tremaroli V. Bäckhed F. Kleerebezem M. (2013). The gut microbiota and mucosal homeostasis: colonized at birth or at adulthood, does it matter? Gut Microbes 4, 118–124. 10.4161/gmic.23362 23333858 PMC3595071

[B13] Faa G. Gerosa C. Fanni D. Nemolato S. van Eyken P. Fanos V. (2013). Factors influencing the development of a personal tailored microbiota in the neonate, with particular emphasis on antibiotic therapy. J. Matern. Fetal Neonatal Med. 26, 35–43 10.3109/14767058.2013.829700 24059551

[B14] Fenton T. R. Kim J. H. (2013). A systematic review and meta-analysis to revise the Fenton growth chart for preterm infants. BMC Pediatr. 13:59. 10.1186/1471-2431-13-59 23601190 PMC3637477

[B15] Fusco V. Quero G. M. Cho G. S. Kabisch J. Meske D. Neve H.. (2015). The genus *Weissella*: taxonomy, ecology and biotechnological potential. Front. Microbiol. 6:155. 10.3389/fmicb.2015.00155 25852652 PMC4362408

[B16] Gough E. K. Stephens D. A. Moodie E. E. Prendergast A. J. Stoltzfus R. J. Humphrey J. H.. (2015). Linear growth faltering in infants is associated with *Acidaminococcus* sp. and community-level changes in the gut microbiota. Microbiome 3:24. 10.1186/s40168-015-0089-2 26106478 PMC4477476

[B17] Hooper L. V. Macpherson A. J. (2010). Immune adaptations that maintain homeostasis with the intestinal microbiota. Nat. Rev. Immunol. 10, 159–169. 10.1038/nri2710 20182457

[B18] Jakobsson H. E. Abrahamsson T. R. Jenmalm M. C. Harris K. Quince C. Jernberg C. (2014). Decreased gut microbiota diversity, delayed Bacteroidetes colonization and reduced Th1 responses in infants delivered by caesarean section. Gut 63, 559–566. 10.1136/gutjnl-2012-303249 23926244

[B19] Kalliomäki M. Collado M. C. Salminen S. Isolauri E. (2008). Early differences in fecal microbiota composition in children may predict overweight. Am. J. Clin. Nutr. 87, 534–538. 18326589 10.1093/ajcn/87.3.534

[B20] Koleva P. T. Bridgman S. L. Kozyrsky A. L. (2015). The infant gut microbiome: evidence for obesity risk and dietary intervention. Nutrients 7, 2237–2260. 10.3390/nu7042237 25835047 PMC4425142

[B21] Ley R. E. Bäckhed F. Turnbaugh P. Lozupone C. A. Knight R. D. Gordon J. I. (2005). Obesity alters gut microbial ecology. Proc. Natl. Acad. Sci. U.S.A. 102, 11070–11075. 10.1073/pnas.0504978102 16033867 PMC1176910

[B22] Scheepers L. E. Penders J. Mbakwa C. A. Thijs C. Mommers M. Arts I. C. (2014). The intestinal microbiota composition and weight development in children: the KOALA birth cohort study. Int. J. Obes. 39, 16–25. 10.1038/ijo.2014.178 25298274

[B23] Schwarzer M. Makki K. Storelli G. Machuca-Gayet I. Srutkova D. Hermanova P.. (2016). *Lactobacillus plantarum* strain maintains growth of infant mice during chronic undernutrition. Science 351, 854–857. 10.1126/science.aad8588 26912894

[B24] Sommer F. Bäckhed F. (2013). The gut microbiota–masters of host development and physiology. Nat. Rev. Microbiol. 11, 227–238. 10.1038/nrmicro2974 23435359

[B25] Vael C. Verhulst S. L. Nelen V. Goossens H. Desager K. N. (2011). Intestinal microflora and body mass index during the first three years of life: an observational study. Gut Pathog. 3:8. 10.1186/1757-4749-3-8 21605455 PMC3118227

[B26] Wang J. Tang H. Wang X. Zhang X. Zhang C. Zhang M.. (2016). The structural alteration of gut microbiota in low-birth-weight mice undergoing accelerated postnatal growth. Sci. Rep. 6:27780. 10.1038/srep27780 27277748 PMC4899793

