# Supplementary material for: Intestinal Microbiota and Weight-Gain in Preterm Neonates
Source: Front Microbiol. 2017 Feb 8;8:183. doi: 10.3389/fmicb.2017.00183 (PMC5296308; doi:10.3389/fmicb.2017.00183)
Supplement: Supplementary file 1 [file Table1.DOC]

Supplementary Table 1. Median and percentiles 25 and 75 [in brackets] obtained for the background characteristics of the population under study.

|  | **Infant age** | | | |
| --- | --- | --- | --- | --- |
|  | ***2 days*** | ***10 days*** | ***30 days*** | ***90 days*** |
| Weight (grams) | 1233 [997-1556] | 1250 [1004-1715] | 1765 [1257-2125] | 3415 [2580-4227] |
| Weight gain (%) | -3.23 [-7.73-0.00] | -2.87 [-9.49-3.18] | 33.11 [17.50-45.18] | 173.91 [132.87-184.32] |
| *Bifidobacterium* (Log nº cells/g) | 5.37 [5.08-5.7] | 5.66 [5.16-6.61] | 6.53 [5.34-8.50] |  |
| *Lactobacillus-group* (Log nº cells/g) | 5.52 [4.48-7.18] | 5.44 [4.83-7.53] | 5.25 [4.09-7.58] |  |
| *Staphylococcus* (Log nº cells/g) | 4.00 [4.00-4.57] | 5.89 [4.63-6.56] | 5.70 [4.54-6.43] |  |
| *Enterococcus* (Log nº cells/g) | 6.46 [6.08-7.00] | 7.97 [6.56-8.50] | 8.16 [7.44-8.69] |  |
| *Bacteroides-group* (Log nº cells/g) | 4.46 [4.03-5.44] | 4.62 [4.25-5.59] | 4.64 [4.25-5.83] |  |
| *Enterobacteriaceae* (Log nº cells/g) | 7.06 [6.04-8.03] | 10.01 [9.42-10.27] | 10.15 [9.58-10.55] |  |
| *Streptococcus* (Log nº cells/g) | 5.00 [4.50-5.98] | 6.97 [5.00-7.59] | 7.49 [6.26-8.09] |  |
| *Weissella* (Log nº cells/g) | 5.68 [4.00-7.71] | 4.99 [4.00-7.72] | 5.00 [4.00-7.45] |  |
| Total bacteria (Log nº cells/g) | 7.06 [6.30-7.74] | 9.82 [8.86-9.92] | 9.89 [9.12-10.39] |  |
| Acetate (µg/ml) | 846.71 [526.2-1113.5] | 2344.46 [1365.4-4942.8] | 3186.17 [2359.2-5310.8] |  |
| Propionate (µg/ml) | 40.78 [0.00-340.30] | 353.30 [137.91-425.66] | 474.04 [355.05-806.60] |  |
| Butyrate (µg/ml) | 0.00 [0.00-7.24] | 0.00 [0.00-85.68] | 184.33 [0.00-312.85] |  |
| Total SCFA (µg/ml) | 863.07 [554.1-1460.6] | 3161.06 [1804.7-5366.8] | 4157.98 [3048.2-6793.2] |  |
