# Supplementary material for: Intestinal Microbiota and Weight-Gain in Preterm Neonates
Source: Front Microbiol. 2017 Feb 8;8:183. doi: 10.3389/fmicb.2017.00183 (PMC5296308; doi:10.3389/fmicb.2017.00183)
Supplement: Supplementary file 2 [file Table2.DOC]

Supplementary Table 2. Levels (Log nº cells/g; mean ± sd) of the different microbial groups analyzed in cesarean section (CS) and vaginally delivered (VD) infants. *Statistically significant differences (Welch test; p<0.05) between CS and VD babies.

|  | ***2 days*** | |  | ***10 days*** | |  | ***30 days*** | |
| --- | --- | --- | --- | --- | --- | --- | --- | --- |
|  | **VD** | **CS** |  | **VD** | **CS** |  | **VD** | **CS** |
| *Bifidobacterium* | 5.41 ± 0.66 | 5.50 ± 0.72 |  | 6.42 ± 1.59 | 5.91 ± 1.26 |  | 6.68 ± 1.58 | 7.00 ± 1.71 |
| *Lactobacillus-*group | 6.23 ± 1.68 | 5.60 ± 1.37 |  | 6.51 ± 1.73 | 6.02 ± 1.60 |  | 5.85 ± 1.88 | 5.86 ± 1.72 |
| *Staphylococcus* | 4.44 ± 0.82 | 4.43 ± 0.87 |  | 5.78 ± 1.52 | 5.69 ± 1.51 |  | 5.43 ± 1.34 | 5.64 ± 1.11 |
| *Enterococcus* | 6.90 ± 1.11 | 6.57 ± 0.96 |  | 7.72 ± 1.00 | 7.63 ± 1.27 |  | 8.04 ± 0.92 | 8.04 ± 0.77 |
| *Bacteroides-*group | 5.05 ± 1.48 | 4.60 ± 0.72 |  | 5.76 ± 1.34 | 4.66 ± 0.87* |  | 5.59 ± 1.43 | 4.83 ± 1.08* |
| *Enterobacteriaceae* | 7.49 ± 1.60 | 7.25 ± 1.54 |  | 10.03 ± 1.33 | 9.69 ± 1.68 |  | 10.2 ± 1.05 | 9.96 ± 0.94 |
| *Streptococcus* | 5.58 ± 1.01 | 5.48 ± 0.75 |  | 6.90 ± 1.61 | 6.55 ± 1.16 |  | 6.90 ± 1.27 | 7.38 ± 1.28 |
| *Weissella* | 6.25 ± 1.57 | 5.75 ± 1.74 |  | 6.13 ± 1.77 | 5.61 ± 1.66 |  | 6.04 ± 1.66 | 5.40 ± 1.64 |
| Total bacteria | 7.47 ± 1.26 | 6.99 ± 1.18 |  | 9.46 ± 0.79 | 9.19 ± 0.99 |  | 9.46 ± 0.70 | 9.43 ± 0.69 |
