# Supplementary figures and images for: Intestinal Microbiota and Weight-Gain in Preterm Neonates
Source: Front Microbiol. 2017 Feb 8;8:183. doi: 10.3389/fmicb.2017.00183 (PMC5296308; doi:10.3389/fmicb.2017.00183)

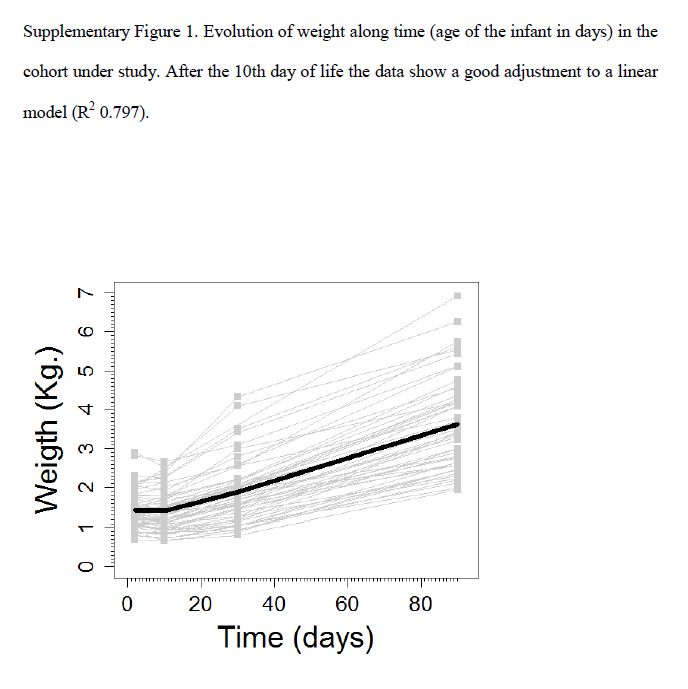

Supplement: Supplementary file 3 [file Image1.JPEG]

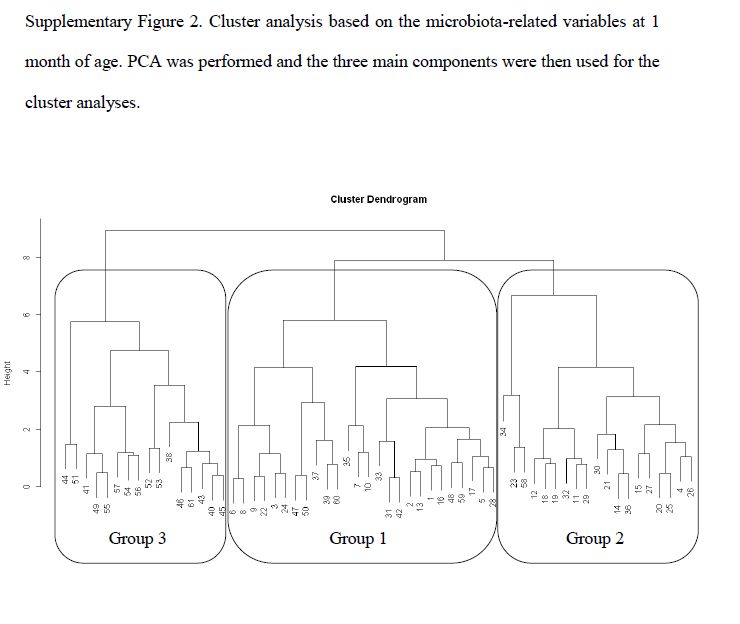

Supplement: Supplementary file 4 [file Image2.JPEG]
